# Lower Hemoglobin Levels as a Risk Factor for the Development of Retinopathy of Prematurity

**DOI:** 10.7759/cureus.64264

**Published:** 2024-07-10

**Authors:** Rama K Gudu, Swaranjika Sahoo, Pravati Jena, Sushree S Behura, Subhadra Priyadarshini, Santosh K Panda

**Affiliations:** 1 Pediatrics, Kalinga Institute of Medical Sciences, Bhubaneswar, IND; 2 Research and Development, Kalinga Institute of Medical Sciences, Bhubaneswar, IND

**Keywords:** neovascularization, hypoxia, anaemia, prevention, prediction, screening, preterm, hemoglobin, neonate, retinopathy of prematurity

## Abstract

Background: Retinopathy of prematurity (ROP) is an important cause of visual morbidity among preterm infants. The objective of the study was to assess the relationship between the initial hematological parameters of the complete blood count (CBC) and ROP development in preterm neonates.

Method: This retrospective cohort study was conducted in a neonatal intensive care unit in Odisha. The hematological parameters of the CBC conducted within the first 48 hours of age, demographic characteristics, neonatal morbidities, and ROP screening findings of preterm neonates (gestational age <34 weeks) were analyzed. Independent risk factors associated with ROP development were identified in a multivariate logistic regression model.

Result: A total of 43 (29.1%) out of 148 neonates had any of the ROP stages (stage 1-26, 2-08, and 3-09). Birth weight (aOR 0.003; 95% CI 0.00, 0.11);hemoglobin (Hb) level (aOR 0.70; 95% CI 0.54, 0.90); presence of respiratory distress syndrome (RDS) (aOR 7.61; 95% CI 1.5, 36.39); and need for packed red blood cell (PRBC) transfusion (aOR 4.26; 95% CI 1.1, 16.44) were independently associated with ROP development. The odds of ROP were higher among the neonates with initial Hb 10.5-15.4 g/dL (OR (95% CI) 3.7(1.5, 8.9), p=0.003) and for neonates with Hb 15.4-17.3 g/dL (OR (95% CI) 2.5(1.01, 6.16), p=0.047) in comparison to neonates with initial Hb >17.3 g/dL.

Conclusion: Preterm neonates with a lower level of Hb during the early postnatal days are at higher risk for ROP development and need to be prioritized for screening.

## Introduction

Retinopathy of prematurity (ROP) is a known morbidity in preterm infants, characterized by the presence of neovascularization in the avascular retina [1]. It is the leading cause of childhood blindness and visual morbidity in preterm infants. Globally, in 2010, around 184,700 preterm babies had any stage of ROP, and 20,000 became blind from it [2]. The prevalence of ROP was around 9.3%-36.6% among very low-birth-weight neonates and/or neonates born < 32 weeks in various nationwide surveys [3]. Over the last two decades, ROP prevalence has increased in developing nations, with a higher survival rate for smaller preterm neonates. It is described as the emergence of the third epidemic of ROP in middle-income countries like India, which needs urgent attention [4]. ROP can be prevented through early prediction, timely screening, and appropriate management. Till now, various demographic characteristics, i.e., lower gestational age, lower birth weight, and neonatal morbidities, i.e., the need for mechanical ventilation, blood transfusion, sepsis, and necrotizing enterocolitis, are established risk factors associated with ROP and help the clinician triage the preterm infants for ROP screening [5]. Considering the burden of ROP, there is always a search for new biomarkers to predict the progression of ROP in preterm infants and strategies for its prevention.

Complete blood count (CBC) parameters are commonly investigated in hospitalized preterm neonates and have a physiological role in disease regulation [6]. Hemoglobin (Hb) is a carrier of oxygen; leukocytes and platelets play an important role in the inflammatory pathway of neonatal diseases [7].In the previous study, the ROP development is associated with the retinal tissue oxygenation state, i.e., hypoxia or hyperoxia, and systemic inflammation [5]. Hence, we hypothesize that there may be an association between alterations in major CBC parameters and the pathway of neovascularization. The objectivity of the study is to assess the relationship between ROP development in preterm <34-week-old neonates and hematological parameters in the CBC obtained during the early postnatal days.

## Materials and methods

This retrospective study was conducted in a tertiary care neonatal unit in India from January 2018 to June 2019, after obtaining institutional ethical permission. 

All preterm babies born <34 weeks gestational age admitted to the neonatal unit within 48 hours of age were included. Neonates who died before ROP screening, were discharged against medical advice before ROP screening, or were lost to follow-up were excluded. 

ROP screening of preterm neonates was conducted as per the national guidelines with parental consent [8]. Neonates born <28 weeks gestational age or birthweight <1,200 g were screened at two to three weeks of age, and neonates with 28-34 weeks of gestational age were screened at four weeks of age. The ROP screening was performed in a neonatal intensive care unit under the supervision of a neonatologist. Neonates discharged prior to the scheduled time of ophthalmological evaluation were screened at a high-risk follow-up clinic.

The trained ophthalmologist of the institute screened the neonates with indirect ophthalmoscopy. Pupils were dilated with phenylephrine 2.5% and tropicamide 0.5%, and a local anesthetic drop was installed prior to eye examination [9]. Comfort care techniques, i.e., administration of oral sucrose solution, nesting, and swaddling, were provided during the screening examination. Retinopathy findings were documented according to the International Classification of Retinopathy of Prematurity (ICROP) Stage [10]. The severe ROP cases were managed with either laser ablation therapy or anti-vascular endothelial growth factor (anti-VEGF) therapy after receiving informed consent from the parents [9]. A follow-up examination was scheduled based on retinal findings as per unit protocol. ROP screening findings were recorded in the ROP register with detailed patient information.

Neonatal demographic profile, i.e., gestational age, birth weight, and mode of delivery, were documented in the predesigned proforma. Gestational age was calculated from the last menstrual period or first trimester ultrasound scan of the mother and postnatal New Ballard scoring. Birth weight was measured by an electronic weighing scale. Neonatal morbidities, i.e., respiratory distress syndrome (RDS), need for mechanical ventilation, hemodynamically significant patent ductus arteriosus (hs-PDA), bronchopulmonary dysplasia (BPD), delayed full feeding, and need for packed red blood cell (PRBC) transfusion, were documented from case records.

The respiratory management, nutrition strategies, and blood component transfusion were based on unit protocol. RDS was diagnosed based on the presence of respiratory distress and a chest X-ray. Preterm neonates with respiratory distress were initiated on continuous positive airway pressure (CPAP), and neonates who failed CPAP were subjected to invasive mechanical ventilation. Surfactant replacement therapy was administered within the first six hours of life by the INSURE (intubation, surfactant administration through an endotracheal tube, and extubating to non-invasive ventilation) method for infants who required FiO_2_ >0.3 after one hour of CPAP support or preterm neonates who were intubated in the delivery room. Hemodynamically significant PDA was labeled with the presence of duct size >1.5 mm and LA/AO ratio >1.4 by bedside echocardiography. Neonates who required more than two weeks to reach enteral feeding of 120 ml/kg/day were labeled as having delayed full feeding. BPD was diagnosed based on the need for oxygen or respiratory support at 28 days of age, 36 weeks postmenstrual age, or hospital discharge [11].

As per unit protocol, the CBC investigations were sent in a 1 ml Ethylene Diamine Tetra-acetic Acid (EDTA) container, either through venipuncture or umbilical central line sampling, at 6-12 hours of age in all inborn neonates and at the time of hospitalization in extramural neonates. The hematological parameters of CBC (collected within the first 48 hours of age) were analyzed in a Coulter counter automated analyzer (Beckman Coulter, LH 780, California) at the institutional central laboratory. The hematological parameters, i.e., Hb, red cell distribution width (RDW), total leucocyte count (TLC), absolute neutrophil count (ANC), absolute lymphocyte count (ALC), absolute monocyte count (AMC), total platelet count (TPC), and mean platelet volume (MPV), were noted. Newer hematological parameters, i.e., neutrophil lymphocyte ratio (NLR), neutrophil monocyte ratio (NMR), and platelet lymphocyte ratio (PLR), were calculated. 

Sample size

In our neonatal unit, about 200 preterm <34 weeks were subjected to ROP screening over the 18-month period preceding this study, and the prevalence of ROP was 30% in a previous study [12]. Assuming population size n = 200, hypothesized percentage frequency of outcome factor (p): 30%, with 95% confidence limits and 5% precision; the estimated sample used was 124. Expecting attrition of 20% among the study population, the final calculated sample size was 148.

Statistical analysis

All quantitative variables were reported as mean (SD) or median (Q1, Q3), and frequency (%) was used for categorical variables. The comparison of neonatal characteristics, morbidities, and hematological parameters of CBC between neonates with ROP and without ROP was done by univariate analysis using an independent t-test or Mann-Whitney U test for continuous data and a chi-square test for categorical data. A multivariate binary logistic regression model was constructed for independent prediction of ROP development. The confounding variables identified by univariate analysis were included as covariate in the multivariate logistic regression model to control for their effect. aOR and 95% CIs were calculated. 

Hb levels were categorized into three groups: less than 25th percentile, 25-50th percentile and greater than 50th percentile. The relative risk (OR) of ROP development for the neonates with Hb levels in the first quartile and second quartile were calculated with respect to the neonates with Hb > 50th percentile. P< 0.05 was considered to be statistically significant. Stata 15.1, Stata Corp., Texas, was used for analysis. 

## Results

During the study period, a total of 167 preterm (<34 weeks) neonates with their initial CBC report within 48 hours of life were included, and a total of 19 neonates were excluded (left against medical advice - 6, neonatal deaths - 11, lost to follow-up - 2). Out of 148 study participants, 43 (29%) neonates had any stage of ROP (26 - Stage 1, 08 - Stage 2, and 09 - Stage 3).

The comparison of neonatal characteristics, hematological parameters, and morbidities between neonates with ROP and those without ROP are enumerated in Table 1. The average gestational age and birth weight of neonates with ROP were significantly lower than those without ROP. When comparing the CBC parameters, lower Hb, TLC, ANC, and ALC and AMC were found in the ROP group in comparison to the no-ROP group. The presence of RDS, invasive mechanical ventilation, BPD, neonatal sepsis, delayed full feeding, and the need for PRBC transfusion were more prevalent in the ROP group than in the no-ROP group.

**Table 1:** Comparison of neonatal demographic characteristics, hematological parameters and morbidities between neonates with and without ROP. ROP - Retinopathy of prematurity; T - t-statistic; U - Mann-Whitney U; χ2 - Chi-square; PRBC: Packed red blood cell; BPD - Bronchopulmonary dysplasia; Hb - Hemoglobin; RDS - Respiratory distress syndrome; TLC - Total leucocyte count; ALC - Absolute lymphocyte count; ANC - Absolute neutrophil count; AMC - Absolute  monocyte count $ - Independent t-test, * -Chi-square test, and # - Mann-Whitney U test are used to compare between ROP vs no ROP. Statistical significance set at a p-value<0.05.

Neonatal characteristics	ROP (n=43)	No ROP (n=105)	T/χ^2^ /U	p-value
Demographic Characteristics				
Gestational age (wk)^$^	28.84±2.15	31.74±2.55	-6.567	<0.001
Birth weight (gm)^$^	1,024±226	1,275±174	-7.271	<0.001
Male*	26 (60.5)	66 (62.9)	0.074	0.785
Caesarean*	17 (39.5)	62 (59.0)	4.667	0.031
Small for gestational age (Yes)*	13 (30.2)	50 (47.6)	4.376	0.089
Antenatal steroid (Yes)*	25 (58.1)	68 (64.8)	0.573	0.460
Haematological Parameters				
Hb (g/dL)^$^	16.32±3.05	17.82±2.55	-3.077	0.002
Red cell distribution width (%)^$^	17.31±2.31	18.04±2.60	-1.604	0.111
TLC^#^	9,100 (6,780-13,660)	12,130 (9,190-16,245)	1,594.5	0.021
ANC^#^	5,104 (3,276-7,870)	6,982 (4,772-10,246)	1,650.0	0.010
ALC^#^	2,894 (2,247-4,736)	3,540 (2,680-5,220)	1,803.0	0.055
AMC^#^	609 (248-867)	759 (451-1,206)	1,687.0	0.016
Neutrophil-lymphocyte ratio^$^	2.16±1.89	2.33±1.49	-0.583	0.561
Neutrophil-monocyte ratio^#^	9.2 (6.7-16.0)	9 (6.3-13.8)	2,088.0	0.474
Total platelet count (Lakh)^$^	2.15±0.78	2.12±0.71	0.235	0.814
Mean platelet volume^$^	9.41±1.57	8.97±1.28	1.739	0.084
Platelet-lymphocyte ratio^#^	65 (40.2-86.0)	57.9 (40.96-69.70)	1,887.0	0.118
Clinical Parameters				
RDS (Yes)^*^	38 (88.4)	41 (39.0)	29.824	<0.001
Surfactant therapy (Yes)^*^	14 (32.5%)	21 (20%)	2.664	0.103
Mechanical ventilation (Yes)^*^	31 (72.1)	59 (39.9)	26.260	<0.001
BPD (Yes)^*^	12 (27.9)	6 (5.7)	14.064	<0.001
Sepsis (Yes)^*^	22 (51.2)	23 (21.9)	12.341	<0.001
Delayed full feeding (Yes)^*^	28 (65.1)	23 (21.9)	25.222	<0.001
PRBC Transfusion (Yes)*	33 (76.7)	21 (20.0)	42.388	<0.001

A multivariate regression model for independent risk factors associated with ROP development is shown in Table 2. Birth weight (aOR 0.003; p = 0.002), Hb level (aOR 0.70; p = 0.006), presence of RDS (aOR 7.61; p = 0.011) and need for PRBC transfusion (aOR 4.26; p = 0.035) were independently associated with ROP development.

**Table 2:** Multivariate regression model for ROP in preterm neonates. PRBC - Packed red blood cell; ROP - Retinopathy of prematurity; BPD - Bronchopulmonary dysplasia; Hb - Hemoglobin; RDS - Respiratory distress syndrome; TLC - Total leucocyte count; ALC: Absolute lymphocyte count; ANC - Absolute neutrophil count; AMC - Absolute  monocyte count Binary logistic regression is used. Statistical significance set at a p-value<0.05.

Neonatal variables	Beta Coefficient	S.E.	p-value	OR	95% CI for OR
Gestational age (weeks)	-0.134	0.171	0.432	0.874	(0.625,1.222)
Birth weight (grams)	-5.737	1.824	0.002	0.003	(0.000,0.115)
Hb	-0.352	0.127	0.006	0.703	(0.548,0.902)
TLC	-0.001	0.001	0.154	0.999	(0.998,1.000)
ANC	0.001	0.001	0.176	1.001	(1.000,1.002)
ALC	0.001	0.001	0.296	1.001	(0.999,1.002)
AMC	0.000	0.001	0.931	1.000	(0.998,1.002)
RDS	2.030	0.798	0.011	7.612	(1.592,36.393)
Mechanical ventilation	-0.090	0.707	0.898	0.914	(0.229,3.653)
BPD	-0.683	0.888	0.442	0.505	(0.089,2.878)
Sepsis	0.004	0.662	0.996	1.004	(0.274,3.674)
Delayed full feeding	-0.162	0.620	0.794	0.851	(0.253,2.865)
PRBC Transfusion	1.449	0.689	0.035	4.260	(1.104,16.443)

The mean (SD) Hb level was significantly lower in neonates with ROP (16.32± 3.05g/dL) in comparison to those without ROP (17.82±2.55g/dL), p = 0.002. Among all neonates, the range of Hb levels was 10.5-23.1 g/dL, and the 25th and 50th percentiles (median) Hb levels were 15.4 g/dL and 17.3 g/dL, respectively. The risk (OR) of ROP development for the neonates with initial Hb 10.5-15.4 g/dL and 15.4-17.3 g/dL in comparison to neonates with initial Hb >17.3 g/dL were OR (95% CI) 3.7 (1.5-8.9), p = 0.003, and OR (95% CI) 2.5 (1.0-6.16), p = 0.047, respectively (Table 3).

**Table 3:** Risk of ROP based on initial Hb level of preterm neonates ROP - Retinopathy of prematurity; Hb - Hemoglobin Data presented as frequency (column %). Statistical significance set at a p-value<0.05.

Hemoglobin (g/dL)	No ROP (n=105)	ROP (n=43)	OR 95% CI	p-value
10.5–15.4	21 (20.0%)	17 (39.5%)	3.7 (1.55–8.97)	0.003
15.4–17.3	24 (22.9%)	13 (30.2%)	2.5 (1.01–6.16)	0.047
>17.3	60 (57.1%)	13 (30.2%)	1.0 (reference)	

## Discussion

In this study, the incidence of ROP was 29% among preterm neonates born before 34 weeks gestational age; and lower birth weight, the presence of lower Hb during the early days, RDS, and the need for PRBC transfusion were independently associated with ROP development.

The incidence of ROP in our study among preterm neonates was similar to previous published studies from India: 30% in a study from central India by Dwivedi et al. and 32% from a southern India by Ahuja et al. [12,13]. It is not surprising that neonates born with a lower gestational age, a lower birth weight, the presence of RDS, and the requirement of PRBC transfusion were at high risk for ROP development, as reported in the majority of studies [5,12,13]. This is the first study to explore the association of lower Hb in early postnatal life with ROP development in a multivariate regression model after adjustment of significant independent variables found in univariate analysis.

In this study, the risk of ROP was higher among neonates with Hb 10.5-15 g/dL (OR 3.7 times) and 15-17 g/dL (OR 2.5 times) in comparison to neonates with Hb >17 g/dL. Though the mean Hb level in ROP group does not fall below anemic cut off level in neonates (<13g/dL), but higher Hb level is protective. However, in two previous studies, lower Hb in preterm neonates in the early days were at higher risk of ROP in only univariate analysis but not as an independent risk factor after adjustment [14,15]. Higher odds of ROP in neonates born with lower Hb <12 gm/dL (OR 3.2), 12-14 gm/dL (OR 4.9), and 14-16 gm/dL (OR 1.9) compared to those neonates born with Hb >18 gm/dL were found in the above study [14]. Similarly, in a study by Lundgren et al., the presence of anemia (<11 g/dL) during the first week of life was an independent predictor of ROP in extremely preterm neonates [16].

Neovascularization of the retina is an important pathophysiology of ROP development [17]. Various angiogenic factors, i.e., VEGF, basic fibroblast growth factor (b-FGF), and hypoxia-induced factors (HIF-1alpha), play an important role in the neovascularization of the retina and are regulated by tissue hypoxia or hyperoxia [17]. Lower Hb levels cause tissue hypoxia and more release of VEGF in the developing retina of preterm infants, which could explain the pathological importance of low Hb and ROP development. Preterm neonates born with higher Hb may have better hemodynamic stability and fewer prematurity-related complications.

Hence, effective intervention in the delivery room for an increment in Hb level immediately via placental transfusion, i.e., delayed umbilical cord clamping or umbilical cord milking, may reduce ROP. However, ROP reduction is not credited to placental transfusion in any systematic review; rather, placental transfusion prevents necrotizing enterocolitis and the need for blood transfusion, which are known risk factors for ROP [18].

This study has its own limitations by including study participants on the gestational age cutoff (<34 weeks) for ROP screening and not considering birth weight criteria. The study is limited to only early CBC parameters and ROP; hence, further studies are needed to evaluate the trend in CBC changes with relation to the development of ROP. Though neonatal respiratory morbidities were considered for ROP development, the detailed information about the need of oxygen therapy, i.e., maximum FiO_2_ requirement, duration of oxygen therapy, duration of hypoxia or hyperoxia of enrolled neonates were not informed.

## Conclusions

We found that presence of a higher Hb level during early postnatal days is protective against the development of ROP in preterm infants. This study emphasizes the implementation of optimum antenatal care to avoid premature delivery, antenatal steroid use to reduce RDS burden, the practice of delayed cord clamping and frequent phlebotomy to avoid iatrogenic anemia, which may reduce ROP development in preterm infants. The presence of anemia at birth may have been secondary to several antenatal comorbidities that may directly or indirectly affect the incidence of ROP, which need to be evaluated in future studies. The study has major implications for middle-income countries, where the third epidemic of ROP has emerged. It could help the clinician triage the preterm infant based on the early Hb level, and neonates with an early lower Hb level are prioritized for ROP screening. 
